# Effect of General Anesthesia vs. Conscious Sedation on the Outcomes of Acute Ischemic Stroke Patients After Endovascular Therapy: A Meta-Analysis of Randomized Clinical Trials

**DOI:** 10.3389/fneur.2019.01131

**Published:** 2019-10-31

**Authors:** Teng-Fei Wan, Jian-Rong Zhang, Liang Liu

**Affiliations:** ^1^Department of First Cadre Ward, The General Hospital of Northern Theater Command, Shenyang, China; ^2^Department of Neurology, Xinqiao Hospital, The Army Military Medical University, Chongqing, China; ^3^Department of Neurology, The General Hospital of Northern Theater Command, Shenyang, China

**Keywords:** acute ischemic stroke, general anesthesia, conscious sedation, endovascular treatment, meta-analysis

## Abstract

**Background:** Endovascular therapy is the standard treatment for acute ischemic stroke (AIS) patients caused by a large vessel occlusion in the anterior circulation, whereas the impacts of general anesthesia (GA) vs. conscious sedation (CS) for such procedures remained as a continued debate.

**Methods:** We systematically searched PubMed, Embase, and ClinicalTrials.gov. We restricted our search to RCTs that examined the clinical outcomes of endovascular therapy with GA vs. CS. The Cochrane Risk of Bias Tool was used to assess study quality. Random-effects or fixed-effects meta-analyses were used for evaluating all outcomes.

**Results:** A total of three randomized clinical trials met our inclusion criteria, with 368 individuals enrolled. Patients were randomized to receive GA or CS during endovascular therapy. In a meta-analysis of these trials, patients in the GA group were associated with favorable functional outcome (mRS score ≤ 2) compared with the CS group (pooled OR = 1.81, 95% CI: 1.17–2.79, *P* = 0.008). Besides, patients in the GA group had higher odds of successful reperfusion (pooled OR = 1.80, 95% CI: 1.05–3.08, *P* = 0.033), but no significant differences were seen in symptomatic intracranial hemorrhage (pooled OR = 0.54, 95% CI: 0.11–2.57, *P* = 0.308), vessel dissection or perforation (pooled OR = 1.38, 95% CI: 0.30–6.31, *P* = 0.679), migration of embolus to a new territory (pooled OR = 2.28, 95% CI: 0.89–5.87, *P* = 0.085), post-operative pneumonia (pooled OR = 1.74, 95% CI: 0.76–4.01, *P* = 0.149), and all-cause mortality at 90 days (pooled OR = 0.73, 95% CI: 0.43–1.26, *P* = 0.263) compared with the CS group.

**Conclusion:** Performing endovascular therapy with GA, compared with CS, improves functional independence after 90 days significantly for patients with AIS caused by a large vessel occlusion in the anterior circulation. However, additional larger and multi-center randomized controlled trials to definitively confirm our findings are warranted for the limitation of the small sample size in this study.

## Background

Acute ischemic stroke (AIS) is one of the leading causes of death and long-term disability. For severe AIS patients caused by large-vessel occlusion in the anterior circulation, several studies have revealed that thrombolytic therapy by intravenous recombinant tissue plasminogen activator (rtPA) in conjunction with endovascular therapy in the form of stent-retriever devices for thrombectomy is more effective than thrombolytic therapy alone (1–3). Moreover, a meta-analysis for eight randomized clinical trials has shown that endovascular therapy with mechanical thrombectomy was associated with improved functional outcomes compared to standard medical care with rtPA for patients with AIS (4). Thus, endovascular therapy was recommended for the treatment of AIS by international guidelines recently (5). However, there are numerous factors that could affect patient outcomes during peri-interventional management of thrombectomy, one of which is anesthetic strategy. In the past decades, several studies have evaluated the impact of anesthesia technique [general anesthesia (GA) or conscious sedation (CS)] on neurological outcome of AIS patients after endovascular therapy (6–9). But results of these trials yielded varied conclusions, warranting further examination. In particular, prior meta-analyses have suggested that AIS patients undergoing endovascular therapy may have worse outcomes when treated with GA compared with CS (10, 11), while some newly randomized controlled trials (RCTs) have conducted and showed conflict results (12–14). Because previous available meta-analysis studies had limitations, such as those studies mainly focused on pooling results from non-randomized and observational studies that were not balanced for baseline parameters and carried risk of bias, a meta-analysis including complete results from recently published RCTs is warranted. To do this, we conduct a systematic review and meta-analysis of RCTs to compare the impact of GA with CS for patients with AIS undergoing endovascular therapy.

## Methods

This systematic review and meta-analysis was conducted using a pre-specified protocol in accordance with the PRISMA (Preferred Reporting Items for Systematic Reviews and Meta-Analyses) statement (15).

### Search Strategy

We searched the related articles in PubMed, Embase, and the clinical trial registry maintained at ClinicalTrials.gov up to November 2018. The keywords we used are the following: *conscious sedation, general anesthesia, endovascular, recanalization, thrombolysis, fibrinolysis, fibrinolytic agents, thromboembolism, catheter, transcatheter, embolectomy*, or *thrombectomy*. This was combined with terms pertinent to the medical condition of interest: *intracranial embolism, thrombosis*, or *stroke*. The references of published reviews and RCTs with potential met our pre-specified inclusion and exclusion criteria and were manually screened to avoid missing any eligible RCTs that were not previously identified. There were no language restrictions. Two investigators (LL and T-FW) independently conducted the literature search.

### Study Selection

Two authors (T-FW and LL) screened the title and abstract of identified publications independently for evaluating studies for eligibility. If discrepancies appeared, which would be resolved with consensus. If necessary, a third reviewer (J-RZ) would be consulted. Inclusion criteria were the following: (1) a randomized clinical trial study design, (2) randomized adult participants (aged ≥ 18 years) with AIS to GA or CS during endovascular therapy, (3) participants with AIS in anterior circulation, (4) reporting of mortality and functional outcome using the modified Rankin scale (mRS) as an end point, and (5) reporting of the effect estimates of studies or calculating the effect estimates from the available data. We excluded case reports, *post-hoc* analyses, observational studies, reviews, editorials, duplicate reports, commentaries, abstracts, animal studies, meeting proceedings, and studies with incomplete information. Moreover, studies not separating outcomes by anesthesia type or participants with posterior circulation stroke were also excluded.

### Data Abstraction

The study and patient characteristics and data on outcomes were abstracted by two authors (T-FW and LL) independently from the trials' primary texts, supplementary appendices, and protocols. Any disagreements were resolved by joint discussion. The study and patient characteristics were extracted including trial name, publication year, trial design type, study period, sources of data, inclusion and exclusion criteria, outcomes, type of endovascular therapy, and sample size in each group. Baseline patient demographics, comorbidities, and treatment-related parameters were also extracted, including sex, age, vascular risk factors, premorbid mRS score (score range: 0–6, with a lower score indicating independent living), admission NIHSS score (score range: 0–42, with higher scores indicating more severe deficits), time interval, occlusion site, intravenous thrombolysis treatment, and hemodynamic and respiratory parameters. Moreover, data on outcomes were then extracted including good functional outcome at 90 days (defined as proportion of patients with an mRS score of 0–2 following endovascular therapy), 90-day mortality, successful reperfusion [thrombolysis in cerebral infarction (TICI) 2b/3], vascular complications [including vessel dissection or perforation, symptomatic intracranial hemorrhage (sICH), migration of embolus to a new territory], and respiratory complications (post-operative pneumonia).

### Quality Assessment

Quality of included RCTs was performed by two investigators (LL and T-FW) independently. The risk of bias for each included RCT was assessed in accordance with the Cochrane Collaboration's tool (16), which include each of the following domains: sequence generation; allocation concealment; blinding of participants, personnel, and outcome assessors; incomplete outcome data; reporting biases; other potential sources of bias. The risk of bias was assigned as a score of low, unclear, or high, in accordance with established criteria. We judged trials as having a moderate risk of bias when the study was with more than two high-risk components. The study with more than four high-risk components was defined as having a high risk of bias, while the study with 0–2 high-risk components was defined as having low risk of bias.

### Statistical Analysis

The primary outcome was good functional outcome at 90 days (defined as patients with an mRS score ≤ 2). The secondary outcomes include successful reperfusion, vascular complications, respiratory complications, and 90-day mortality. Odds ratios (ORs) with their 95% confidence intervals (CIs) were used as a measure of the association of GA with each outcome of interest compared to CS. The random-effects meta-analysis model (DerSimonian-Laird method) or fixed-effects meta-analysis model (Mantel-Haenszel method) was used to pool count data across trials and the statistical significance of pooled ORs and 95% CIs were determined with an equivalent *Z* test (17). Which model should be used for pooling count data across trials was in accordance with the heterogeneity among the included RCTs. The heterogeneity among the RCTs included in our meta-analysis was assessed by the *P*-value of chi-square-based *Q* tests and the *I* squared (*I*^2^) statistic. As previous studies reported, the *I*^2^ value was <50% and the *P*-value of the *Q* test was more than 0.1 among the RCTs included in the meta-analysis, which may suggest no obvious heterogeneity across studies. Then, the fixed-effects model was used for pooling across studies, while the *I*^2^ values of more than 50% and the *P*-value of the *Q* test of <0.1 may indicate the studies included in the meta-analysis with obvious heterogeneity. Then, the random-effects model was used (18). Statistical analyses were conducted using STATA software, version 12.0 (StataCorp, College Station, TX, USA). Statistical significance was set to *P* < 0.05.

## Results

### Study Selection and Study Characteristics

A total of 368 potentially relevant publications were identified (Figure S1). Of these, 102 duplicated records and 273 unrelated records were excluded after screening by title and abstract. Of the remaining 24 publications for full-text review, 21 studies were excluded for not meeting our pre-specified inclusion criteria and only three trials were eligible in our meta-analysis (12–14) (Figure S1): Sedation vs. Intubation for Endovascular Stroke Treatment (SIESTA), Sedation vs. General Anesthesia for Endovascular Therapy in Acute Stroke—Impact on Neurological Outcome (AnStroke), and General Or Local Anesthesia in Intra Arterial THerapy (GOLIATH). All eligible studies were single-center, parallel-group, open-label RCTs with blinded end point evaluation, and the results of those studies were published between 2016 and 2018. All included RCTs had a maximum follow-up of 90 days after the procedure. Locations of ischemic strokes among included patients were all confirmed by CT/MRI scan within the anterior circulation distribution. The 90-day mRS score was one of the outcomes for all included RCTs. In AnStroke trial, 90-day mRS score was the primary outcome, but in SIESTA and GOLIATH, it was the second outcome. The characteristics of these included RCTs are summarized in Table 1. Among these three RCTs, a total of 368 individuals were enrolled and randomized to receive GA or CS during endovascular therapy, including 183 patients with GA and 185 receiving CS. The distributions of patient characteristics were similar across studies, including demographics and clinical characteristics (Table 2).

**Table 1:** Characteristics of the randomized clinical trials included in the meta-analysis.

**Trials**	**Study design**	**Study period**	**Country**	**Inclusion criteria**	**Exclusion criteria**	**Outcomes**	**Type of Endovascular Treatment**	**Sample Size (GA/CS)**
SIESTA, 2016 (12)	RCT	2014–2016	Germany	Age ≥18 years; anterior circulation AIS; occlusion of carotid artery and/or middle cerebral artery; NIHSS > 10; planned mechanical recanalization; informed consent from patient or legal representative	Age <18 years; informed consent not obtainable; coma; agitation; vomiting; difficult airway management; additional cerebral hemorrhage	Primary outcomes: NIHSS score after 24 h. Secondary outcomes: mRS and mortality rate after 3 month; inpatient-mortality	Stent, mechanical thrombectomy	73/77
AnStroke, 2017 (13)	RCT	2013–2016	Sweden	Age ≥18 years; anterior circulation AIS, NIHSS score ≥10 (if right-sided occlusion) or ≥14 (if left-sided occlusion); groin puncture started <8 h after symptom onset	Patient was not eligible for randomization for the anesthesiological concerns; posterior circulation stroke; intracerebral hemorrhage; neurological recovery or recanalization before or during angiography; premorbidity mRS score ≥ 4 or other comorbidity contraindicating embolectomy	Primary outcomes: mRS after 3 months. Secondary outcomes: NIHSS score at days 3, 7, and 90; TICI scores at day 1; periprocedural complications; infarct growth	Mechanical thrombectomy	45/45
GOLIATH, 2018 (14)	RCT	2015–2017	Denmark	Age ≥18 years; NIHSS > 10; mRS ≤ 2; groin puncture started <6 h from stroke onset; occlusion of ICA, ICA-T, M1, and M2	MRI contraindications; GCS <9; intubated prior to arrival; posterior circulation stroke; allergy to anesthetics	Primary outcome: infarct growth; Secondary outcomes: mRS scores after 90 days, time, and blood pressure levels, and safety end points	Stent, mechanical thrombectomy	65/63

*GA, general anesthesia; CS, conscious sedation; RCT, randomized controlled trial; AIS, acute ischemic stroke; NIHSS, National Institutes of Health Stroke Scale; mRS, modified Rankin Scale; TICI, thrombolysis in cerebral infarction; ICA, internal carotid artery; MRI, magnetic resonance imaging; GCS, Glasgow Coma Scale*.

**Table 2:** Baseline patient characteristics among included randomized clinical trials.

**Characteristics**	**SIESTA, 2016** **(****12****)**		**AnStroke, 2017** **(****13****)**		**GOLIATH, 2018** **(****14****)**	
	**General anesthesia (*n* = 73)**	**Conscious sedation (*n* = 77)**	**General anesthesia (*n* = 45)**	**Conscious Sedation (*n* = 45)**	**General Anesthesia (*n* = 65)**	**Conscious Sedation (*n* = 63)**
Age, mean (SD), or median (IQR), years	71.8 (12.9)	71.2 (14.7)	73 (65–80)	72 (66–82)	71.0 (10.0)	71.8 (12.8)
Women, *n* (%)	25 (34.2)	35 (45.5)	19 (42.2)	22 (48.9)	29 (44.6)	33 (52.4)
**Vascular risk factors**						
Hypertension, *n* (%)	53 (72.6)	54 (70.1)	27 (60.0)	22 (48.9)	39 (60.0)	32 (50.8)
Atrial fibrillation, *n* (%)	36 (49.3)	36 (46.8)	18 (40.0)	18 (40.0)	24 (36.9)	27 (42.9)
Diabetes, *n* (%)	17 (23.3)	17 (22.1)	9 (20.0)	7 (15.6)	9 (13.8)	9 (14.3)
Smokers, *n* (%)	9 (12.3)	13 (17.1)^a^	4 (8.9)	8 (17.8)	20 (30.8)	20 (31.7)
Hyperlipidemia, *n* (%)	20 (27.4)	24 (31.2)	5 (11.1)	7 (15.6)	NS	NS
Premorbid mRS score ≤ 2, *n* (%)	64 (87.7)	71 (92.2)	44 (97.7)	44 (97.7)	63 (96.9)	63 (100.0)
Admission NIHSS score, median (IQR)	17 (13–20)	17 (14–20)	20 (15.5–23)	17 (14–20.5)	18 (13–21)	17 (15–21)
ASPECTS score, median (IQR)	8 (7–9)	8 (6.25–9)	10 (8–10)	10 (9–10)	NS	NS
**Occlusion site**, ***n*** **(%)**						
Internal carotid artery	1 (1.4)	9 (11.7)	15 (33.3)	10 (22.2)	14 (21.5)	13 (20.6)
M1 MCA	39 (53.4)	43 (55.8)	26 (57.7)	26 (57.7)	21 (32.3)	32 (50.8)
M2 MCA	7 (9.6)	4 (5.2)	0 (0)	8 (17.7)	12 (18.5)	7 (11.1)
Intravenous thrombolysis treatment, *n* (%)	46 (63.0)	50 (64.9)	33 (73.3)	36 (80.0)	50 (76.9)	46 (73.0)

*SD, standard deviation; IQR, interquartile range; NIHSS, National Institutes of Health Stroke Scale; ASPECTS, Alberta Stroke Program Early CT (computed tomography) Score; MCA, middle cerebral artery*.

a *Percent is based on a denominator of 76 patients*.

## Quality Assessment

The quality of all eligible studies was rated as low risk of bias (Table S1), as assessed by the Cochrane Risk of Bias Tool. All of the eligible studies had a high risk of bias in the blinding of participants, personnel, and outcome assessors category, because these RCTs were open-label trials, which were blinded for outcome assessors but not researchers and participants.

### Outcomes

**Figure 1:**
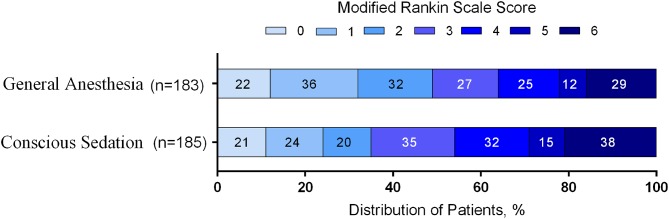
Functional outcome at 90-day follow-up of GA vs. CS. The modified Rankin scale measures functional outcome on a seven-point ordinal scale: 0, no symptoms at all; 1, no significant disability despite symptoms; 2, slight disability; 3, moderate disability; 4, moderately severe disability; 5, severe disability; 6, death.

The distribution of mRS scores separated by trial is reported in Table 3. Moreover, a graphical summary of the seven scores of the mRS between both GA and CS groups at 90 days was pooled and is shown in Figure 1. Individual and pooled 90-day favorable outcome (mRS score ≤ 2) is presented in Table 4. The results of our meta-analysis suggested that patients randomized to endovascular therapy with GA have significantly higher rates of favorable functional outcome at 90 days (mRS score ≤ 2) compared with CS (pooled OR = 1.81, 95% CI: 1.17–2.79, *P* = 0.008).

**Table 3:** Distribution of 90-day modified Rankin scale scores by treatment group.

**Modified Rankin scale score**	***n*** **(%)**					
	**SIESTA, 2016** **(****12****)**		**AnStroke, 2017** **(****13****)**		**GOLIATH, 2018** **(****14****)**	
	**General anesthesia (*n* = 73)**	**Conscious sedation (*n* = 77)**	**General anesthesia (*n* = 45)**	**Conscious sedation (*n* = 45)**	**General anesthesia (*n* = 65)**	**Conscious sedation (*n* = 63)**
0	3 (4.1)	4 (5.2)	7 (15.6)	10 (22.3)	12 (18.5)	7 (11.1)
1	11 (15.1)	7 (9.1)	6 (13.3)	3 (6.7)	19 (29.2)	14 (22.2)
2	13 (17.8)	3 (3.9)	6 (13.3)	5 (11.1)	13 (20.0)	12 (19.1)
3	9 (12.3)	21 (27.2)	10 (22.3)	6 (13.3)	8 (12.3)	8 (12.7)
4	12 (16.4)	17 (22.1)	6 (13.3)	5 (11.1)	7 (10.8)	10 (15.9)
5	7 (9.6)	6 (7.8)	4 (8.9)	5 (11.1)	1 (1.5)	4 (6.3)
6	18 (24.7)	19 (24.7)	6 (13.3)	11 (24.4)	5 (7.7)	8 (12.7)

**Table 4:** Summary of pooled analyses for primary and secondary outcomes.

**Outcomes**	**SIESTA, 2016** **(****12****)**		**AnStroke, 2017** **(****13****)**		**GOLIATH, 2018** **(****14****)**		**Meta-analysis results**		
	**General anesthesia (*n* = 73)**	**Conscious sedation (*n* = 77)**	**General anesthesia (*n* = 45)**	**Conscious sedation (*n* = 45)**	**General anesthesia (*n* = 65)**	**Conscious sedation (*n* = 63)**	**OR (95% CI)**	***P*-value**	***I^**2**^* (%)**
**Primary outcome**									
Modified Rankin Scale 0–2 after 90 days, *n* (%)	27 (37)	14 (18.2)	19 (42.2)	18 (40.0)	31 (47.7)	21 (33.3)	1.81 (1.17–2.79)	0.008	14.8
**Secondary outcomes**									
Successful reperfusion (TICI 2b/3), *n* (%)	65 (89.0)	62 (80.5)	41 (91.1)	40 (88.9)	50 (76.9)	38 (60.3)	1.80 (1.05–3.08)	0.033	0
Vessel dissection or perforation, *n* (%)	1 (1.4)	2 (2.6)	3 (6.7)	1 (2.2)	0 (0)	0 (0)	1.38 (0.30–6.31)	0.679	10.1
sICH, *n* (%)	NA	NA	0 (0)	3 (6.7)	2 (3.1)	1 (1.6)	0.54 (0.11–2.57)	0.308	15.3
Migration of embolus to a new territory, *n* (%)	NA	NA	5 (11)	1 (2.2)	10 (15.4)	6 (9.5)	2.28 (0.89–5.87)	0.085	0
Post-operative pneumonia, *n* (%)	10 (13.7)	3 (3.9)	6 (13.3)	7 (15.6)	NA	NA	1.74 (0.76–4.01)	0.194	65.7
Mortality at 90 days, *n* (%)	18 (24.7)	19 (24.7)	6 (13.3)	11 (24.4)	5 (7.7)	8 (12.7)	0.73 (0.43–1.26)	0.263	0

*OR, odds ratio; CI, confidence interval; TICI, thrombolysis in cerebral infarction; sICH, symptomatic intracranial hemorrhage; NA, not applicable*.

Among the subgroup analyses of successful reperfusion (TICI 2b/3), we found that the GA group was significantly higher than the CS group (pooled OR = 1.80, 95% CI: 1.05–3.08, *P* = 0.033; Table 4). The occurrence of anesthesiological or interventional complications is presented in Table 4. Pooling the results from the fixed-effects or random-effects model showed that GA was not associated with increased risk of anesthesiological or interventional complications, including vessel dissection or perforation (pooled OR = 1.38, 95% CI: 0.30–6.31, *P* = 0.679), sICH (pooled OR = 0.54, 95% CI: 0.11–2.57, *P* = 0.308), migration of embolus to a new territory (pooled OR = 2.28, 95% CI: 0.89–5.87, *P* = 0.085), and post-operative pneumonia (pooled OR = 1.74, 95% CI: 0.76–4.01, *P* = 0.149). In addition, there was no significant difference in mortality rate at 90 days between GA and CS groups (pooled OR = 0.73, 95% CI: 0.43–1.26, *P* = 0.263; Table 4). The intraoperative variables including the time intervals and hemodynamic and respiratory data are shown in Table S2.

No significant differences were observed across trials between the GA and CS groups in early neurological outcomes as measured by the median (interquartile range, IQR) 24-h NIHSS score change (Table S3). The SIESTA trial reported that the mean difference in decline at 24 h (adjusted for baseline NIHSS) between the groups was not statistically significant (GA vs. CS: −5 [−10 to 2] vs. −4 [−10 to 2]; *P* = 0.82). The NIHSS score shifts at 24 h were also similar for both groups in the AnStroke trial (GA vs. CS: 9 (4–17) vs. 8 [2.5–13], *P* = 0.27). In the GOLIATH trial, 24-h change in NIHSS score favored GA over CS but also was not statistically significant (GA vs. CS: −10 [−14 to −5] vs. −7 [−13 to 0]; *P* = 0.11). All eligible studies have measured final infarct size after the procedure, and no statistically significant difference was found between the GA and CS groups in both SIESTA and AnStroke trials, while the GOLIATH trial has observed that final infarct volume was smaller in the GA group (median [IQR], GA vs. CS: 22.3 [8.1–64.5] ml vs. 38.0 [16.7–128.0] ml; *P* = 0.04) (Table S3). Besides, only the GOLIATH trial has reported the end point of infarct growth and showed no statistically significant difference between the GA and CS groups (median [IQR], GA vs. CS: 8.2 [2.2–38.6] ml vs. 19.4 [2.4–79.0] ml; *P* = 0.10) (Table S3).

## Discussion

This study was designed to analyze all of the recently published randomized clinical trials comprehensively, which compared GA vs. CS during endovascular therapy for patients with AIS. We found that patients with GA undergoing endovascular therapy showed higher rate of good functional outcome at 90 days. In addition, compared with CS, GA was associated with higher rate of successful reperfusion during endovascular therapy, but showed no significant differences in mortality at 90 days, anesthesiological or interventional complications, including vessel dissection or perforation, sICH, migration of embolus to a new territory, and post-operative pneumonia. These findings provide increased precision concerning GA as a viable anesthetic approach during endovascular therapy.

In contrast, previous systematic reviews and meta-analyses showed that AIS patients undergoing endovascular therapy have worse outcomes when treated with GA as compared with CS (10, 11, 19, 20). However, these meta-analysis pooled data mainly from observational and non-randomized trials where the choice of CS or GA for a given AIS patient was not randomized. Thus, the results of these meta-analyses may be confounded by indication and selection bias since GA was often chosen for the patients with more severe illness. For example, in the GA group, the patients with worse outcomes were driven mainly by those who had a medical indication for GA in a *post-hoc* analysis of the IMS III trial (21). Furthermore, Badhiwala et al. reported that stent retrievers were associated with more favorable outcomes than other devices (4). Thus, another new meta-analysis conducted by Adeel Ilyas and colleagues (22) pooled the results from nine trials in which patients performed endovascular therapy by modern thrombectomy devices, including stent retriever devices and/or a direct aspiration, and found that the use of either GA or CS during endovascular therapy did not yield significant difference in the functional independence at 90 days. But these findings mainly relied on observational and non-randomized trials. To eliminate the influence of the type of mechanical device used for endovascular thrombectomy on revascularization and functional outcomes, all included trials in our meta-analysis mainly used stent retriever devices and/or a direct aspiration. Conversely, a meta-analysis of individual patient data from seven RCTs, including MR CLEAN (1), ESCAPE (23), EXTEND-IA (24), SWIFT PRIME (2), REVASCAT (25), PISTE (26), and THRACE (27) trials, showed worse outcomes after endovascular thrombectomy was associated with GA (28). However, the primary purpose of these included RCTs was to examine the association between endovascular mechanical thrombectomy and clinical outcomes among patients with AIS, but not intent to evaluate the effects of anesthetic strategy on clinical outcomes for patients with AIS undergoing endovascular therapy. Besides, the design of these trials was also not randomized by the choice of CS or GA for a given AIS patient. To the best of our knowledge, our meta-analysis was the first to pool recent published RCTs concerning which anesthetic strategy results in the best clinical outcomes undergoing endovascular therapy. Importantly, among these RCTs, the choice of CS or GA was randomized undergoing endovascular therapy.

The findings of our meta-analysis firstly reflect that patients treated under GA did not associate with poorer outcomes compared with CS, but improved functional outcomes at 90 days were seen among patients in the GA group. These results may be due to the higher rate of recanalization among patients in the GA group. Both in SIESTA and GOLIATH trials, the higher rate of successful reperfusion (TICI 2b/3) was found in the GA group but not in the AnStroke trial (12–14). This may explain why the AnStroke trial did not show a benefit in functional outcome among GA group. These data may indicate that endovascular therapy under GA is likely associated with greater technical success such as providing complete patient immobility. Indeed, we must acknowledge that this result should be interpreted with caution, because many literatures vary on this point and found the rates of recanalization was similar between GA and CS (29, 30). As Simonsen et al. have mentioned (14), this conflict may be explained by the differences in institutional or operator experience with performing endovascular therapy using CS.

Furthermore, other factors that may influence the association between anesthesia and outcomes after endovascular therapy should be of concern, such as hemodynamic disturbance, treatment delay, and inhaled anesthetic agents. As reported in several studies, during the acute phase of stroke, a small decrease (20–30 mmHg) in blood pressure has been associated with worse outcomes (31–34). Consistently, intraoperative blood pressure was less in the GA group and was associated with less favorable outcomes in a *post-hoc* analysis of the MR CLEAN trial (35). But there was no formal protocol specifying blood pressure targets in the MR CLEAN trial, and about 75% of the patients had a systolic blood pressure lower than 140 mmHg in this trial. In contrast, among the included trials in our meta-analysis, the specified criteria have been made to maintain systolic blood pressure at more than 140 mmHg throughout the endovascular therapy in SIESTA, AnStroke, and GOLIATH trials. Thus, even though a lower intraoperative blood pressure was shown in the GA group compared with CS in both AnStroke (mean [95% CI], GA vs. CS: 144.9 [141.3; 148.0] vs. 147.2 [144.0; 150.4]) and GOLIATH trials (mean [SD], GA vs. CS: 143 (15) vs. 155 (20)), patients in the GA group did not present less favorable outcomes (13, 14). These data therefore indicated that maintaining systolic blood pressure at an appropriate threshold (more than 140 mmHg) may be essential for patients undergoing endovascular therapy with GA.

We must acknowledge that this study has several potential limitations. First, the major limitation of this study is the small sample size; only three high-quality trials with a total of 368 patients were included in this meta-analysis, which may limit the study power to find the clinically relevant differences in outcome. Second, we were only able to get part of the data among the included trials. Some of the baseline characteristics were not available. Thus, we could not conduct some subgroup analysis, such as by baseline NIHSS score, intraoperative blood pressure, and time to treatment. Lastly, all included trials in this meta-analysis were conducted at a single center in Europe; thus, the quality of a body of evidence may be moderate. For these reasons, some multi-center RCTs conducted in other countries or continents have been sponsored, such as the Choice of ANesthesia for EndoVAScular Treatment of Acute Ischemic Stroke (CANVAS) trial in China (NCT02677415) (36). Despite these limitations, our study represents the best available evidence regarding the effect of GA vs. CS on the outcomes of AIS patients after endovascular therapy.

## Conclusions

Our study was designed to compare the effect of GA vs. CS on the outcomes of AIS patients after endovascular therapy. The pooled data from our meta-analysis of RCTs suggested that performing endovascular therapy with GA, compared with CS, is not associated with worse clinical outcomes for patients with AIS in the anterior circulation. Furthermore, the use of GA during endovascular therapy showed higher rate of successful reperfusion and favorable outcomes at 90 days for patients with AIS in the anterior circulation as long as severe hypotension is avoided. However, these findings are limited by the small sample size. Thus, additional larger sample size and multi-center RCTs to definitively confirm our findings is warranted.

## Data Availability Statement

The datasets used and/or analyzed in the present study are available from the corresponding author on reasonable request.

## Author Contributions

LL conceived the study. T-FW and J-RZ collected the data and drafted the manuscript. LL revised the manuscript and language.

### Conflict of Interest

The authors declare that the research was conducted in the absence of any commercial or financial relationships that could be construed as a potential conflict of interest.

## Abbreviations

aICH: Asymptomatic intracranial hemorrhage
AIS: Acute ischemic stroke
AnStroke: Sedation vs. General Anesthesia for Endovascular Therapy in Acute Stroke–Impact on Neurological Outcome
CANVAS: Choice of ANesthesia for EndoVAScular Treatment of Acute Ischemic Stroke
CI: Confidence interval
CS: Conscious sedation
FE: Fixed effects
GA: General anesthesia
GOLIATH: General Or Local Anesthesia in Intra Arterial Therapy
IA: Intra-arterial
IV: Intra-venous
mRS: Modified Rankin scale
NIHSS: National Institutes of Health Stroke Scale
OR: Odds ratio
PRISMA: Preferred Reporting Items for Systematic Reviews and Meta-Analyses
RCT: Randomized controlled trial
RE: Random effects
sICH: Symptomatic intracranial hemorrhage
SIESTA: Sedation vs. Intubation for Endovascular Stroke Treatment
TICI: thrombolysis in cerebral infarction
tPA: Tissue plasminogen activator.

## References

[1] Berkhemer OA Fransen PS Beumer D van den Berg LA Lingsma HF Yoo AJ. A randomized trial of intraarterial treatment for acute ischemic stroke. N Engl J Med. (2015) 372:11–20. 10.1186/1471-2407-9-42 25517348

[2] Saver JL Goyal M Bonafe A Diener HC Levy EI Pereira VM. Stent-retriever thrombectomy after intravenous t-PA vs. t-PA alone in stroke. N Engl J Med. (2015) 372:2285–95. 10.1056/NEJMoa1415061 25882376

[3] Goyal M Menon BK van Zwam WH Dippel DW Mitchell PJ Demchuk AM. Endovascular thrombectomy after large-vessel ischaemic stroke: a meta-analysis of individual patient data from five randomised trials. Lancet. (2016) 387:1723–31. 10.1016/S0140-6736(16)00163-X 26898852

[4] Badhiwala JH Nassiri F Alhazzani W Selim MH Farrokhyar F Spears J. Endovascular thrombectomy for acute ischemic stroke: a meta-analysis. JAMA. (2015) 314:1832–43. 10.1001/jama.2015.13767 26529161

[5] Powers WJ Derdeyn CP Biller J Coffey CS Hoh BL Jauch EC. 2015 American Heart Association/American Stroke Association focused update of the 2013 guidelines for the early management of patients with acute ischemic stroke regarding endovascular treatment: a guideline for Healthcare Professionals From the American Heart Association/American Stroke Association. Stroke. (2015) 46:3020–35. 10.1161/STR.0000000000000074 26123479

[6] Abou-Chebl A Lin R Hussain MS Jovin TG Levy EI Liebeskind DS. Conscious sedation versus general anesthesia during endovascular therapy for acute anterior circulation stroke: preliminary results from a retrospective, multicenter study. Stroke. (2010) 41:1175–9. 10.1161/STROKEAHA.109.574129 20395617

[7] Abou-Chebl A Zaidat OO Castonguay AC Gupta R Sun CH Martin CO. North American SOLITAIRE Stent-Retriever Acute Stroke Registry: choice of anesthesia and outcomes. Stroke. (2014) 45:1396–401. 10.1161/STROKEAHA.113.003698 24668201

[8] Just C Rizek P Tryphonopoulos P Pelz D Arango M. Outcomes of general anesthesia and conscious sedation in endovascular treatment for stroke. Canad J Neurol Sci. (2016) 43:655–58. 10.1017/cjn.2016.256 27406422

[9] Bekelis K Missios S Mackenzie TA Tjoumakaris S Jabbour P Anesthesia technique and outcomes of mechanical thrombectomy in patients with acute ischemic stroke. Stroke. (2017) 48:361–6. 10.1161/STROKEAHA.116.015343 28070000 PMC5263179

[10] Brinjikji W Murad MH Rabinstein AA Cloft HJ Lanzino G Kallmes DF. Conscious sedation versus general anesthesia during endovascular acute ischemic stroke treatment: a systematic review and meta-analysis. Am J Neuroradiol. (2015) 36:525–9. 10.3174/ajnr.A4159 25395655 PMC8013063

[11] Jing R Dai HJ Lin F Ge WY Pan LH. Conscious sedation versus general anesthesia for patients with acute ischemic stroke undergoing endovascular therapy: a systematic review and meta-analysis. BioMed Res Int. (2018) 2018:2318489. 10.1155/2018/2318489 29789778 PMC5896359

[12] Schönenberger S Uhlmann L Hacke W Schieber S Mundiyanapurath S Purrucker JC. Effect of conscious sedation vs general anesthesia on early neurological improvement among patients with ischemic stroke undergoing endovascular thrombectomy a randomized clinical trial. JAMA. (2016) 316:1986–96. 10.1001/jama.2016.16623 27785516

[13] Lowhagen Henden P Rentzos A Karlsson JE Rosengren L Leiram B Sundeman H General anesthesia versus conscious sedation for endovascular treatment of acute ischemic stroke: the anstroke trial (anesthesia during stroke). Stroke. (2017) 48:1601–7. 10.1161/STROKEAHA.117.016554 28522637

[14] Simonsen CZ Yoo AJ Sørensen LH Juul N Johnsen SP Andersen G. Effect of general anesthesia and conscious sedation during endovascular therapy on infarct growth and clinical outcomes in acute ischemic stroke: a randomized clinical trial. JAMA Neurol. (2018) 75:470–7. 10.1001/jamaneurol.2017.4474 29340574 PMC5885172

[15] Moher D Liberati A Tetzlaff J Altman DG. Preferred reporting items for systematic reviews and meta-analyses: the PRISMA statement. BMJ. (2009) 339:b2535. 10.1136/bmj.b2535 19622551 PMC2714657

[16] Higgins JP Altman DG Gøtzsche PC Jüni P Moher D Oxman AD. The cochrane collaboration's tool for assessing risk of bias in randomised trials. BMJ. (2011) 343:d5928. 10.1136/bmj.d5928 22008217 PMC3196245

[17] Borenstein M Higgins JP. Meta-analysis and subgroups. Prev. Sci. (2013) 14:134–43. 10.1007/s11121-013-0377-7 23479191

[18] Higgins JP Thompson SG Deeks JJ Altman DG. Measuring inconsistency in meta-analyses. BMJ. (2003) 327:557–60. 10.1136/bmj.327.7414.557 12958120 PMC192859

[19] Brinjikji W Pasternak J Murad MH Cloft HJ Welch TL Kallmes DF. Anesthesia-related outcomes for endovascular stroke revascularization: a systematic review and meta-analysis. Stroke. (2017) 48:2784–91. 10.1161/STROKEAHA.117.017786 28904228

[20] Ouyang F Chen Y Zhao Y Dang G Liang J Zeng J. Selection of patients and anesthetic types for endovascular treatment in acute ischemic stroke: a meta-analysis of randomized controlled trials. PloS one. (2016) 11:e0151210. 10.1371/journal.pone.0151210 26953574 PMC4783038

[21] Abou-Chebl A Yeatts SD Yan B Cockroft K Goyal M Jovin T. Impact of general anesthesia on safety and outcomes in the endovascular arm of interventional management of stroke (IMS) III trial. Stroke. (2015) 46:2142–8. 10.1161/STROKEAHA.115.008761 26138125 PMC4519363

[22] Ilyas A Chen CJ Ding D Foreman PM Buell TJ Ironside N. Endovascular mechanical thrombectomy for acute ischemic stroke under general anesthesia versus conscious sedation: a systematic review and meta-analysis. World Neurosurg. (2018) 112:e355–67. 10.1016/j.wneu.2018.01.049 29355808

[23] Goyal M Demchuk AM Menon BK Eesa M Rempel JL Thornton J. Randomized assessment of rapid endovascular treatment of ischemic stroke. N Engl J Med. (2015) 372:1019–30. 10.1056/NEJMoa1414905 25671798

[24] Campbell BC Mitchell PJ Kleinig TJ Dewey HM Churilov L Yassi N. Endovascular therapy for ischemic stroke with perfusion-imaging selection. N Engl J Med. (2015) 372:1009–18. 10.1056/NEJMoa1414792 25671797

[25] Jovin TG Chamorro A Cobo E de Miquel MA Molina CA Rovira A. Thrombectomy within 8 hours after symptom onset in ischemic stroke. N Engl J Med. (2015) 372:2296–306. 10.1056/NEJMoa1503780 25882510

[26] Muir KW Ford GA Messow CM Ford I Murray A Clifton A. Endovascular therapy for acute ischaemic stroke: the Pragmatic Ischaemic Stroke Thrombectomy Evaluation (PISTE) randomised, controlled trial. J Neurol Neurosurg Psychiatry. (2017) 88:38–44. 10.1136/jnnp-2016-314117 27756804 PMC5256149

[27] Bracard S Ducrocq X Mas JL Soudant M Oppenheim C Moulin T. Mechanical thrombectomy after intravenous alteplase versus alteplase alone after stroke (THRACE): a randomised controlled trial. Lancet Neurol. (2016) 15:1138–47. 10.1016/S1474-4422(16)30177-6 27567239

[28] Campbell BCV van Zwam WH Goyal M Menon BK Dippel DWJ Demchuk AM. Effect of general anaesthesia on functional outcome in patients with anterior circulation ischaemic stroke having endovascular thrombectomy versus standard care: a meta-analysis of individual patient data. Lancet Neurol. (2018) 17:47–53. 10.1016/S1474-4422(17)30407-6 29263006

[29] Jumaa MA Zhang F Ruiz-Ares G Gelzinis T Malik AM Aleu A. Comparison of safety and clinical and radiographic outcomes in endovascular acute stroke therapy for proximal middle cerebral artery occlusion with intubation and general anesthesia versus the nonintubated state. Stroke. (2010) 41:1180–4. 10.1161/STROKEAHA.109.574194 20431082

[30] Slezak A Kurmann R Oppliger L Broeg-Morvay A Gralla J Schroth G. Impact of anesthesia on the outcome of acute ischemic stroke after endovascular treatment with the solitaire stent retriever. Am J Neuroradiol. (2017) 38:1362–7. 10.3174/ajnr.A5183 28473340 PMC7959912

[31] Sandset EC Bath PM Boysen G Jatuzis D Kõrv J Lüders S. The angiotensin-receptor blocker candesartan for treatment of acute stroke (SCAST): a randomised, placebo-controlled, double-blind trial. Lancet. (2011) 377:741–50. 10.1016/S0140-6736(11)60104-9 21316752

[32] John S Thebo U Gomes J Saqqur M Farag E Xu J. Intra-arterial therapy for acute ischemic stroke under general anesthesia versus monitored anesthesia care. Cerebrovasc. Dis. (2014) 38:262–7. 10.1159/000368216 25401730

[33] Mistry EA Mistry AM Nakawah MO Khattar NK Fortuny EM Cruz AS. Systolic blood pressure within 24 hours after thrombectomy for acute ischemic stroke correlates with outcome. J Am Heart Assoc. (2017) 6:e006167. 10.1161/JAHA.117.006167 28522673 PMC5524120

[34] Bu X Li C Zhang Y Xu T Wang D Sun Y. Early blood pressure reduction in acute ischemic stroke with various severities: a subgroup analysis of the CATIS trial. Cerebrovasc Dis. (2016) 42:186–95. 10.1159/000444722 27110711 PMC8784237

[35] Treurniet KM Berkhemer OA Immink RV Lingsma HF Ward-van der Stam VMC Hollmann MW. A decrease in blood pressure is associated with unfavorable outcome in patients undergoing thrombectomy under general anesthesia. (2018) 10:107–11. 10.1136/neurintsurg-2017-012988 28404769

[36] Peng Y Li Y Jian M Liu X Sun J Jia B. Choice of ANesthesia for EndoVAScular Treatment of acute ischemic stroke: protocol for a randomized controlled (CANVAS) trial. Int. J. Stroke. (2017) 12:991–7. 10.1177/1747493017706243 28436307

