# Supplementary material for: Effect of General Anesthesia vs. Conscious Sedation on the Outcomes of Acute Ischemic Stroke Patients After Endovascular Therapy: A Meta-Analysis of Randomized Clinical Trials
Source: Front Neurol. 2019 Oct 31;10:1131. doi: 10.3389/fneur.2019.01131 (PMC6834687; doi:10.3389/fneur.2019.01131)
Supplement: Supplementary file 1 [file Table_1.DOCX]

**Effect of General Anesthesia versus Conscious Sedation on the Outcomes of Acute Ischemic Stroke Patients after Endovascular Therapy: A Meta-analysis of Randomized Clinical Trials**

**Supplementary Online Content**

**Figure S1.** Flowchart of Literature Search and Study Selection

**Table S1.** Assessment of the Methodological Quality of Included Randomized Trials Using the Cochrane Collaboration’s Tool

**Table S2.** Intraoperative Variables of GA vs CS

**Table S3**. Early Neurological Outcomes of GA vs CS

**Figure S1.** Flowchart of Literature Search and Study Selection


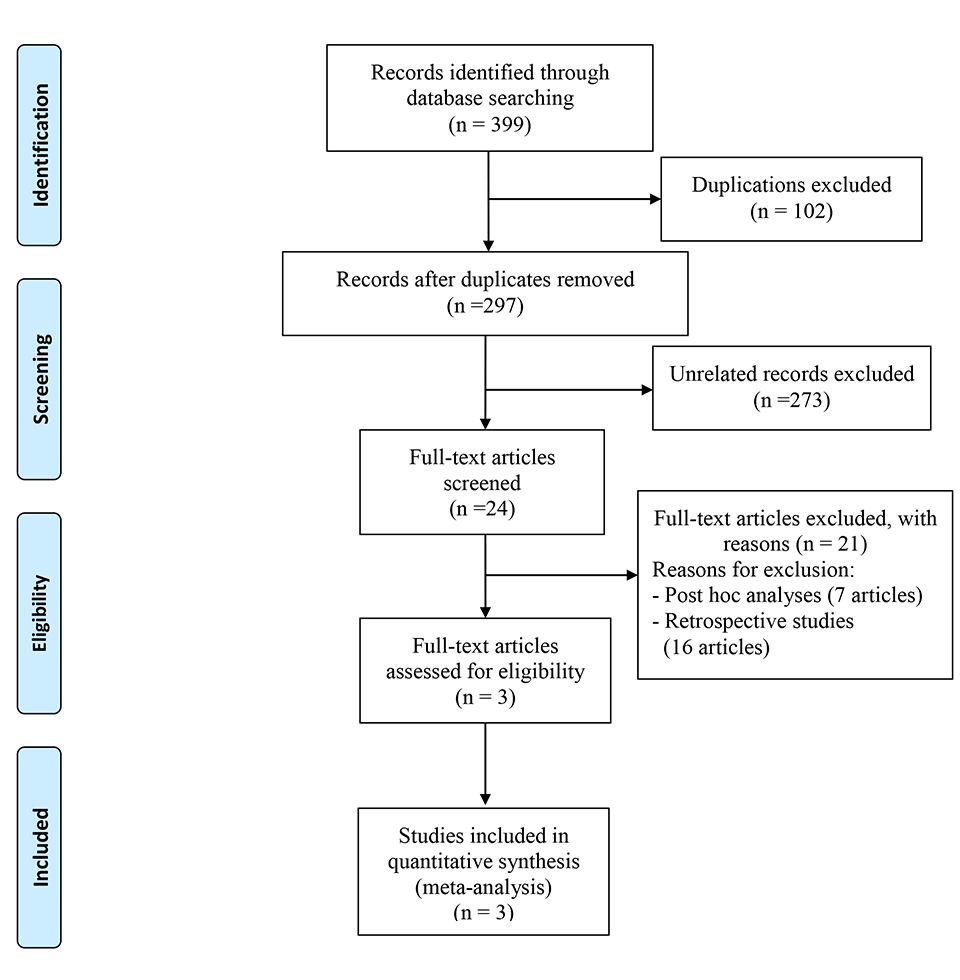


**Table S1.** Assessment of the Methodological Quality of Included Randomized Trials Using the Cochrane Collaboration’s Tool

| **Trials** | Sequence generation | Allocation concealment | Blinding of participants, personnel and outcome assessors | Incomplete outcome data | Selective outcome reporting | Other sources of bias |
| --- | --- | --- | --- | --- | --- | --- |
| SIESTA, 2016 | Low | Low | High | Low | Low | Low |
| AnStroke, 2017 | Low | Low | High | Low | Low | Low |
| GOLIATH, 2018 | Low | Low | High | Low | Low | Low |

**Table S2**. Intraoperative Variables of GA vs CS

| Variables | SIESTA, 2016 | | AnStroke, 2017 | | GOLIATH, 2018 | |
| --- | --- | --- | --- | --- | --- | --- |
|  | General Anesthesia (n = 73) | Conscious Sedation (n = 77) | General Anesthesia (n = 45) | Conscious Sedation (n = 45) | General Anesthesia (n = 65) | Conscious Sedation (n = 63) |
| **Time Interval** |  |  |  |  |  |  |
| Onset-to-door time, mean (SD) or median (IQR), min | 145.0 (83.8) | 118.1 (61.5) | 97 (62–160) | 72 (58–119) | 118 (77.5-203.8) | 112 (68.5-185.0) |
| Time from arrival at neurointerventional suite to groin puncture, median (IQR), min | NA | NA | 34 (18–47) | 25 (15–36) | 24 (20-27) | 15 (12-20) |
| Time from onset to groin puncture, mean (SD) or median (IQR), min | NA | NA | 183 (135–279) | 180 (137–252) | 202 (71) | 186 (72) |
| Time from imaging to groin puncture, median (IQR), min | NA | NA | 92 (68–121) | 91 (55–123) | 61 (48-73) | 54 (40-75) |
| Time from groin puncture to reperfusion/end of procedure, mean (SD) or median (IQR), min | 111.6 (62.5) | 129.9 (62.5) | 55 (38–110) | 74 (37–104) | 34 (21-51) ^a^ | 29 (16-51) ^b^ |
| Time from onset to reperfusion, median (IQR), min | 174.4 (56.3) | 165.2 (59.4) | 254 (206–373) | 250 (213–316) | 212 (180-288) ^a^ | 216 (162-285) ^b^ |
| **Hemodynamic and respiratory data** |  |  |  |  |  |  |
| Baseline MAP before induction of anesthesia, mean (SD), mmHg | NA | NA | 105 (16) | 108 (17) | 110 (17.0) | 108 (18.4) |
| Maximum MAP during the procedure, mean (SD), mm Hg | NA | NA | 116 (15) | 114 (15) | 120 (16) | 117 (19) |
| Minimum MAP during the procedure, mean (SD), mm Hg | NA | NA | 68 (12) | 77 (15) | 72 (13) | 86 (16) |
| Mean systolic blood  pressure during the intervention, mean (95% CI) or mean (SD), mmHg | 144.9  (141.3; 148.0) | 147.2  (144.0; 150.4) | NA | NA | 143 (15) | 155 (20) |
| Mean oxygen saturation during the intervention, mean (95% CI), or mean (SD),% | 98.6 (98.3; 98.9) | 98.2 (97.9; 98.6) ^c^ | 98 (1) | 96 (2) | NA | NA |

Abbreviations: SD, standard deviation; IQR, interquartile range; NA, not applicable; MAP, CI, confidence interval.

^a^ For the 50 reperfused patients.

^b^ For the 38 reperfused patients.

^c^ 75 For the general anesthesia group.

**Table S3.** Early Neurological Outcomes of GA vs CS

| Outcomes | SIESTA, 2016 | | AnStroke, 2017 | | GOLIATH, 2018 | |
| --- | --- | --- | --- | --- | --- | --- |
|  | General Anesthesia (n = 73) | Conscious Sedation (n = 77) | General Anesthesia (n = 45) | Conscious Sedation (n = 45) | General Anesthesia (n = 65) | Conscious Sedation (n = 63) |
| Change in NIHSS after 24 h, median (IQR) | −5 (−10 to 2) | −4 (−10 to 2) | 9 (4 to 17) | 8 (2.5 to 13) | −10 (−14 to −5) | −7 (−13 to 0) |
| Final infarct volume, median (IQR) or mean (95% CI), mL | 60.4 (39.9; 80.9) ^a^ | 69.9 (51.0;88.8) ^b^ | 20 (10–100) | 20 (9–55) | 22.3 (8.1-64.5) | 38.0 (16.7-128.0) |
| Infarct volume growth, median (IQR), mL | NA | NA | NA | NA | 8.2 (2.2-38.6) | 19.4 (2.4-79.0) |

Abbreviations: NIHSS, National Institutes of Health Stroke Scale; IQR, interquartile range; CI, confidence interval; NA, not applicable.

^a^ 68 For the general anesthesia group.

^b^ 73 For the conscious sedation group.
